# Human Decidual Stromal Cells as a Component of the Implantation Niche and a Modulator of Maternal Immunity

**DOI:** 10.1155/2016/8689436

**Published:** 2016-04-28

**Authors:** Kameliya Vinketova, Milena Mourdjeva, Tsvetelina Oreshkova

**Affiliations:** Department of Molecular Immunology, Institute of Biology and Immunology of Reproduction, Bulgarian Academy of Sciences, 73 Tsarigradsko Shose, 1113 Sofia, Bulgaria

## Abstract

The human decidua is a specialized tissue characterized by embryo-receptive properties. It is formed during the secretory phase of menstrual cycle from uterine mucosa termed endometrium. The decidua is composed of glands, immune cells, blood and lymph vessels, and decidual stromal cells (DSCs). In the process of decidualization, which is controlled by oestrogen and progesterone, DSCs acquire specific functions related to recognition, selection, and acceptance of the allogeneic embryo, as well as to development of maternal immune tolerance. In this review we discuss the relationship between the decidualization of DSCs and pathological obstetrical and gynaecological conditions. Moreover, the critical influence of DSCs on local immune cells populations as well as their relationship to the onset and maintenance of immune tolerance is described.

## 1. Human Decidual Development and Structure

**Figure 1:**
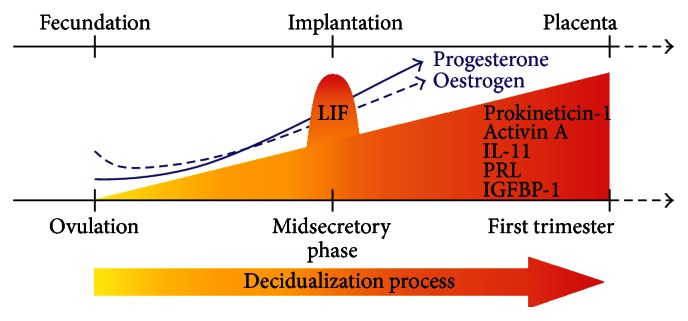
Temporal representation of the decidualization process starting after the ovulation phase of the menstrual cycle and progressing through the first trimester of pregnancy. Progesterone and oestrogen levels increase until the end of gestation, while the decidualization factors, prokineticin-1, activin A, IL-11, PRL, and IGFBP-1 increase in the first trimester until the complete formation of the placenta. LIF is a decidualization factor peaking at peri-implantation.

*(1) Contribution of the Maternal Decidua to Placental Development*. The success of human pregnancy strongly depends on embryo quality and the physiological state of the uterine lining—an epithelial tissue layer called endometrium. To prepare the uterus for embryo implantation and pregnancy, the endometrium undergoes a process termed decidualization. During this process, the endometrial epithelium, blood vessels, and stroma are transformed into a specialized tissue, called decidua [1, 2]. The decidualization process initiates during the midsecretory phase of the menstrual cycles as a result of the elevated levels of ovarian hormones—oestrogen and progesterone [3–5]—independent of the presence of an implanting blastocyst (Figure 1). It causes a gradual and profound alteration in gene expression, cellular functions, and tissue remodelling until the complete formation of a placenta during pregnancy. Analyses of gene expression and secretome of the decidua reveal changed profiles of signal messengers/intermediates, transcription factors, hormones/growth factors, cytokines, chemokines, adhesion molecules, ligands/receptors, cytoskeleton organization, composition of extracellular matrix, ion and water transport, cell cycle regulation, cell trafficking, migration and functions, angiogenesis, decidual receptivity, and implantation [6–10]. The reported magnitude of changes in gene expression altogether suggests an important turning point at the start of the decidual transformation. Initially triggered by the oestrogen or/and progesterone, all components of the functional endometrial layer, that is, glandular epithelium, endometrial stromal cells (ESCs), and endothelium, respond by interrelated and simultaneous activation of multiple factors and mediators. Some factors such as the progesterone receptors (PRs) [11, 12] and the angiogenic factors—VEGF [13, 14], FGF [15], and prokineticin-1 [16]—have oestrogen or progesterone response elements in their promoter region suggesting direct regulation. However, the decidualization-related markers such as tissue factor (TF) [17, 18], insulin-like growth factor binding protein-1 (IGFBP-1) [19], prolactin (PRL) [20], and leukemia inhibitory factor (LIF) [21] do not have hormone response elements in their promoter regions. Nevertheless, their transcription is enhanced by hormones, and this effect is mediated by the activation of signalling pathways, transcription factors, and coactivators (Figure 1).

PR expression also plays a role in the signalling of stimuli maintaining endometrial homeostasis during preparation for pregnancy. PRs are nuclear receptors that exist in two isoforms (PR-A and PR-B) and have different functions [11, 22]. They are stimulated by oestrogen [11, 12] and therefore show the highest levels of expression in the proliferative stage of the menstrual cycle [22]. Furthermore, during the postovulatory rise of progesterone, PRs are gradually downregulated by their ligand (progesterone) even in the presence of oestrogen [12, 22, 23]. In the first trimester of pregnancy, the amount of PR is further decreased with a remaining expression of PR-A predominantly in stromal cells [22]. Nevertheless, PRs are present throughout pregnancy [24] with prevailing PR-B isoform at term [25]. Altogether, the expression level of PR in the human decidua is the net result of a complex regulation by oestrogen, progesterone, and prostaglandins [25] and autoregulation of its own promoter region [26]. The physiological significance of the gradual decrease of PR expression in the endometrium as decidualization advances and pregnancy begins [22, 27] might imply intrinsic subsiding of PR signalling, limiting the response to progesterone [28]. PR response elements are found in the promoter regions of a number of factors. Among them are PRL [28] and IGFBP-1 [29, 30] that experience inhibitory effect, while extracellular matrix component fibronectin [31] is positively influenced by the signalling via PR response elements. The existing regulation between the steroid hormones and their receptors presumably provides counteracting signals that drive decidual transformation and maintain the mechanisms of endometrial homeostasis.

Experimental data strongly indicates that progesterone alone is a weak inductor of decidualization in human ESC as evaluated by the synthesized PRL [28] and IGFBP-1 [20]. However,* in vivo* progesterone exerts endocrine control on the differentiating endometrium and immune cells together with oestrogen [5]. Oestrogen does not induce decidualization on its own [32, 33], but when added to progesterone and incubated for a prolonged period of time (longer than 8 days), it leads to an increase in PRL [34, 35] and IGFBP-1 levels in ESCs [9]. Altogether, progesterone and oestrogen, deprived of decidual environment, are not strong stimulators of decidualization. This implies that other hormones, relaxin [20, 36, 37] and corticotropin-releasing factor (CRF) [38, 39]; decidualization factors, IL-11 [34, 35], activin A (a member from the transforming growth factor beta superfamily) [33, 40], IL-6 [38], and LIF [32]; and prostaglandin E2 (PGE2) [37, 41, 42] from the endometrial niche synergistically augment decidual transformation of ESCs as measured by PRL and IGFBP-1.

**Figure 2:**
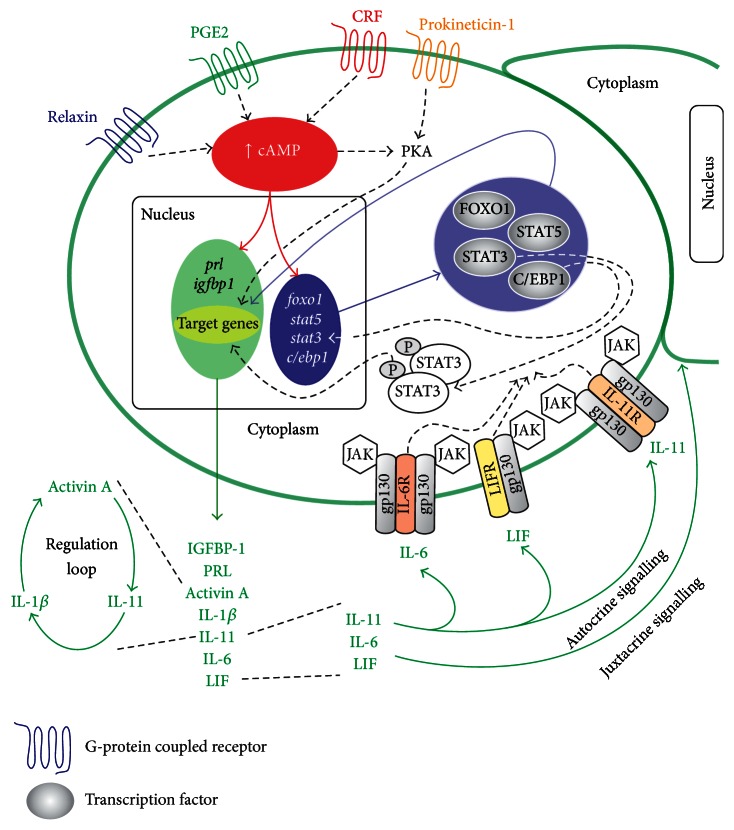
Targets of cAMP signalling during decidualization.

It is well documented that the ovarian hormones progesterone [41], estradiol [43], and relaxin [36], as well as CRF [39] and PGE2 [41, 44], induce accumulation of intracellular cAMP. cAMP is synthesized from adenosine triphosphate via the activation of the enzyme adenylate cyclase [43, 44] and signals via the protein kinase A (PKA) pathway [45]. It is a second messenger in the cells and induces the synthesis of essential factors/morphogens, some of them not directly regulated by progesterone. In combination with progesterone and estradiol, cAMP provides synergistic enhancement of decidualization [28, 39] and induces the synthesis of IL-11 [37], LIF [21], activin A [33, 46], PRL [36], IGFBP-1 [47], and others (Figure 2). These secretory factors, produced in the epithelial and stromal cells of the endometrium, are considered to act in an autocrine and paracrine manner and sequentially activate genes that control the morphological and functional changes associated with decidual differentiation, implantation, trophoblast proliferation/invasion, and recruitment of immune cells.

Prokineticin-1 [16, 48], TF [49], activin A [50, 51], IL-11 [52, 53], PRL [53], and IGFBP-1, which are known decidualization factors, increase in the epithelial and stromal cells of the endometrium starting in the secretory phase and usually increasing in the first trimester of pregnancy (Figure 1). The only exception from this group is LIF, which peaks at the midsecretory/luteal phase [32, 53] in accordance with the expected implantation of the blastocyst [48, 54]. Depending on the function of the aforementioned factors, they might either be indispensable for the induction and maintenance of decidualization or be a result of the process. However, abrogation of endometrial differentiation using inhibitors/neutralization binding proteins, antagonists, knock-down approaches, neutralizing antibodies, or signalling inhibitors against activin A [33, 46], LIF [32], prokineticin-1 [55], and IL-11 [35, 37] shows the crucial role of each of these factors in the decidualization process. Importantly, successful decidualization is critical for the establishment of pregnancy and it is qualitatively and quantitatively evaluated by the amounts of produced PRL and IGFBP-1 [56].

All factors and hormones that are upregulated at the onset of decidualization characteristically have pleiotropic function. The interdependence between them reveals simultaneous, alternative, and sequential manner of activation. For example, prokineticin-1, a protein induced by progesterone, oestrogen, and human chorionic gonadotropin (hCG) [16, 48, 57], stops epithelial cell proliferation, potentiates decidualization [55], and increases angiogenesis and endothelial permeability [58]. Therefore, it is considered that prokineticin-1 contributes to the processes of implantation and placentation during pregnancy [59]. Its action in implantation is mediated by the induction of factors such as cyclooxygenase 2, PGE2, IL-6, IL-11, and LIF [7, 48, 52]. The latter three are members of the IL-6 family of cytokines and share a common signalling chain (gp130) of their receptors [60]. IL-11 and LIF increase the expression of adhesion molecules and the attachment of endometrial epithelial cells to fibronectin and collagen IV [54]. In particular, IL-11 augments the adhesion of endometrial epithelial cells to primary trophoblasts [54], while LIF upregulates adhesion molecules in trophoblasts [61] and increases the adhesion of trophoblasts to fibronectin and laminin (components of the extracellular matrix) [48]. Therefore, the increased adherence of trophectoderm (trophoblasts) to the epithelial cells of the decidua facilitates the attachment and implantation of the blastocyst. In further stages of placental development, trophoblast migration and invasion are mediated by IL-11 [62, 63] and LIF [61].

IL-11 regulation is an excellent example of the complexity of the operational network during decidualization. IL-11 is an important cytokine involved in decidualization [35], implantation [54], and placentation [62]. It is stimulated alternatively or by convergence of signalling pathways of prokineticin-1 [52], activin A [40], relaxin, and PGE2 [37], known as early inducers of decidualization. Relaxin and PGE2 stimulate IL-11 via cAMP/PKA [37] and prokineticin-1 via calcineurin-NFAT signalling pathways [52]. The elevated cAMP/PKA activates IL-11 by the intermediary implication of activin A (Figure 2). This sequence of activation is demonstrated by the usage of activin A receptor inhibitor that downregulates IL-11 production [40]. Activin A, on the other hand, is activated by its own network of factors including progesterone, IL-1*β*, cAMP, and CRF [46, 64–66]. However, IL-1*β* is also a downstream target of IL-11 [67] and seems to form an autoregulatory loop via the activation of activin A. IL-1*β* is secreted in low levels by decidual cells [67] as well as by human preimplantation embryos [68] and promotes decidualization and implantation [2]. Altogether the sequential activation of activin A, IL-11, IL-1*β*, and PRL factors [37, 40, 67] only partially depicts the complex program of decidual transformation, and deficiency in its fulfilment might abrogate or aggravate the state of pregnancy.

*(2) Signalling Pathways Controlling Decidualization*. The receptors of prokineticin-1, relaxin, PGE2, and CRF, like the chemokines' receptors, are G-protein coupled receptors characterized by the presence of seven transmembrane spanning domains [52, 56]. They activate adenylate cyclase that consequently forms the second messenger cAMP. cAMP signalling may be conveyed either by direct influence on genes possessing cAMP responsive elements in their promoter regions (e.g., PRL [36] and IGFBP-1 [47]) or by the activation of protein kinase A (PKA) [37, 56, 69] (Figure 2). As mentioned earlier, cAMP shortens the time and amplifies the magnitude of the decidualization response of endometrial cells. cAMP acts downstream of progesterone, as antiprogestin treatment inhibits cAMP-induced decidualization [56]. cAMP induces the expression of the transcription factors FOXO1 (forkhead/winged helix protein O1A) [70], signal transducers and activators of transcription (STATs) [71], p53, and CCAAT/enhancer-binding protein *β* (C/EBP*β*) and further regulates target genes [8]. Analyses of tissue samples confirm significant upregulation of FOXO1 and C/EBP*β* from the midsecretory phase of the cycle [70, 72, 73]. When applied simultaneously with cAMP, progesterone has an additive effect on FOXO1 expression [72], suggesting that it amplifies the effect of signals from the decidual microenvironment. FOXO1 is the main transcription factor that dominates and controls cell differentiation and represses cell cycle regulation genes during decidualization [8]. It cooperatively interacts with other transcription factors such as C/EBP*β* and PR [8] and regulates the expression of PRL [70] and IGFBP-1 [30].

The synchronization of pregnancy-related tissue development and differentiation implies that cytokines and peptide growth factors and hormones act in a network. The majority of the factors are engaged in transduction signalling via STAT3 or STAT5. STATs are phosphorylated by cytokines receptor-associated Janus kinases (JAK) or by Src family kinases [71]; they dimerize, translocate to the nucleus, and activate target genes [74]. Suppressors of cytokine signalling (SOCS), which are specifically induced by cytokine stimuli, counteract STATs activation, thus forming a negative feedback regulatory mechanism.

Decidualization stimuli such as oestrogen, progesterone, and cAMP specifically upregulate STAT3 [75, 76] and STAT5 [71] in endometrial stromal cells.* In vivo* STAT3 is maximally expressed in glandular epithelium during the secretory phase and in decidual cells during late-secretory phase [76]. Phosphorylated STAT3 is the activated form of the bulk STAT3 and is the convergent focus of LIF [32], IL-11 [76], and IL-6 [77] cytokine signalling. Moreover, STAT3 is a downstream target of the transcription factor C/EBP*β* [78] demonstrating the crosstalk of signalling pathways in cells (Figure 2).

The engagement of STAT3 in endometrial differentiation is indispensable. This has been demonstrated by pathway inhibition approaches with progesterone receptor antagonists, SOCS3 [76], and small interfering/silencing RNA (siRNA) [78]. These treatments cause a significant reduction of PRL and IGFBP-1 production leading to defective decidualization. However, other studies show enhancement of PRL and IGFBP-1 expression [79] via activation of STAT5 signalling [71]. The STAT5 pathway is also an important signal for the decidualization programme of endometrial cells. In summary, STAT3 and STAT5 have been shown to be simultaneously involved into decidual cell differentiation because inhibition of their signalling activity abrogates decidualization.

Altogether, attaining the state of endometrial competence and receptivity is a process regulated by steroid hormones and multiple growth factors. Various signalling pathways are active; they interconnect, converge, diverge, overlap, and amplify to ensure the inherent progress of pregnancy.

**Figure 3:**
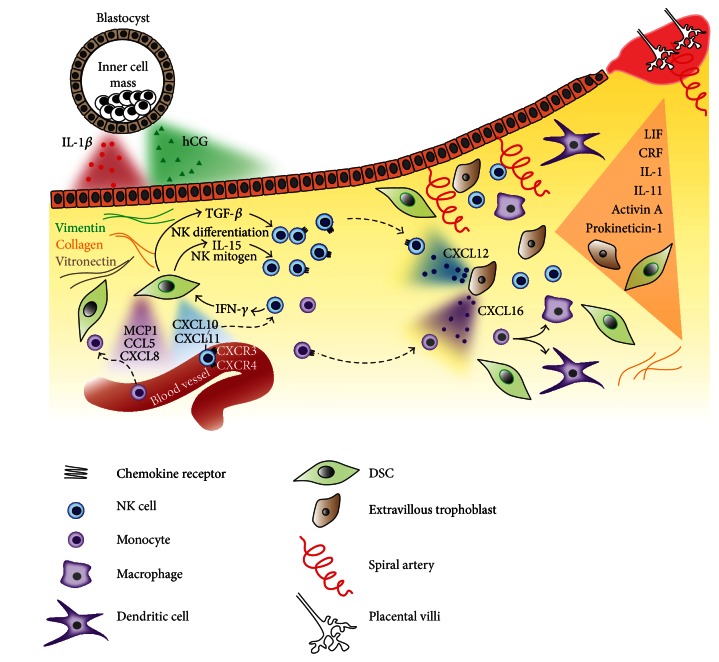
Cell recruitment, invasion, and spatial distribution and communication of foetal and maternal cells in the pre- and postimplantation tissue reorganization during placental formation. Decidual transformation of maternal endometrium is induced by ovary hormones and secretory factors released by the embryo. Recruitment of immune cells is performed by chemokines secreted from the differentiating endometrial and trophoblast cells. Immune cell maintenance/proliferation and differentiation are regulated by the cytokines and factors from endometrial and trophoblast cells creating a prompting microenvironment.

*(3) The Embryonic Trophectoderm Contributes to Placental Development*. The decidualization is a prerequisite for endometrial receptivity. It is essential but not sufficient for the implementation of implantation. Therefore, signalling from the blastocyst is necessary for completing the hormone-driven differentiation of endometrium. A functional crosstalk established via paracrine action of hormones and growth factors synchronizes the development of the preimplantation embryo and endometrial decidualization. Viable early blastocysts secrete hCG [80, 81] starting from day 7 after insemination [82]. At this early stage, the blastocysts are often still in their preimplantation period of development [83]. Investigation of the values of hCG, oestrogen, and progesterone in blood samples from fertile women in conceptive and nonconceptive cycles revealed a correlative enhancement of the ovarian estradiol and progesterone with hCG levels in pregnant women during preimplantation period compared to lower estradiol and progesterone levels in nonpregnant women [84]. After adhesion, the blastocysts significantly augment the secretion of hCG suggesting an important role for trophoblast attachment in differentiation and the establishment of pregnancy [82]. Physiologically, the implantation of the blastocyst into the maternal decidua occurs during a limited time window of 6–10 days after the surge of luteinizing hormone, which marks the ovulation [83, 85]. Thereafter, the embryonic trophectoderm releases hCG in the maternal serum and urine [83, 86], indicating that the embryo has successfully breached the decidual lining and has established contact with the maternal blood. After implantation, during the first 10–12 weeks of prenatal development the embryos secrete increasing amounts of hCG, which subsequently decline in the second and third trimester [87]. This temporal distribution underlines the importance of hCG for the establishment of viable pregnancy and the development of placental tissue. In particular, some of the reported functions of hCG relate to sustaining elevated levels of ovarian hormones [84], stimulating decidualization factors, and mediating trophoblast invasion as shown in* in vitro* models [88]. Furthermore, some cellular and molecular mechanisms of the foetal-maternal dialogue are attributed to hCG specifically released by the embryo. This hormone interacts with its receptors on epithelial cells of the maternal endometrium and successively induces the expression of prokineticin-1 and LIF [48]. As mentioned previously, hCG, prokineticin-1, and LIF are members of the intricate network of factors that exert autocrine and/or paracrine stimulation on the epithelial and stromal cells and influence endometrial decidualization and receptivity. Moreover, CRF [89], prokineticin-1 [59], IL-11 [62], activin A [90], LIF [91], and IL-1 [92, 93] are also secreted by the trophoblast cells or preimplantation embryos, which highlights the contribution of the embryo to the ongoing process of decidual transformation in the stages before and after implantation. The pleiotropic nature of these hormones and cytokines is further revealed by the observation that they participate in the sequence of events leading to embryo acceptance. These include apposition and adhesion of the blastocyst to the extracellular matrix, breaching of the epithelial basal lamina, and the invasion of the endometrial stroma by the trophectoderm [94, 95]. The interaction of the embryo with the decidua is mediated via the expression of integrins [96, 97]—heterodimeric transmembrane glycoproteins which mediate cell-to-cell or cell-to-substratum adhesion by binding to cellular ligands and proteins from the extracellular matrix [85]. In this manner, the decidua and in particular the stromal cells are primed by decidualization factors and secrete extracellular matrix molecules such as collagens, laminin, and vitronectin [98, 99] that participate in tissue remodelling (Figure 3). Among several integrins regulated during menstrual cycle [97], the *α*_v_ *β*_3_ integrin, which is expressed by endometrial epithelial and stromal cells, is defined as a marker for uterine receptivity [85, 93]. In fertile women it is upregulated during the implantation window when the endometrium becomes receptive [97, 100, 101]. Besides steroid hormones [102], *α*_v_ *β*_3_ is stimulated by IL-1*α* and IL-1*β* secreted by human embryos, showing that embryo can regulate endometrial receptivity [93]. Known ligands of *α*_v_ *β*_3_ are fibronectin, fibrinogen, vitronectin, von Willebrand, osteopontin, and collagen [85]. Some studies show that trophoblasts are able to secrete collagen [103], while others demonstrate LIF-mediated enhancement of trophoblast adhesion to fibronectin and laminin [48]. These data suggest the existence of active reciprocal mechanisms for strengthening embryo attachment to the maternal decidua. These mechanisms are controlled by hormones and factors such as hCG, prokineticin-1, and LIF, and their effect is to enhance receptivity and maintain early pregnancy [48].

*(4) Placenta*. The collaboration of the endometrium and trophectoderm constitutes the endocrine component of the placenta that maintains the course of pregnancy [104]. Both tissues synthesize the same hormones and factors, such as CRF [89, 104], prokineticin-1 [52, 59], LIF [21, 53, 91], IL-11 [53, 62], and activin A [65, 66, 90] (Figure 3), while at the same time they differentially express the corresponding receptors [48, 59, 62, 89, 105–107]. Crucially, this set of factors attains reciprocal responsiveness, regulation, and synchronization of tissue remodelling during placentation. As a result, in a healthy pregnancy, the placenta develops only after an equilibrated exposure to maternal and foetal factors, cells, and tissues. Crucially, the maternal immune system intervenes in the processes of placentation and ensures the proper implementation of tissue remodelling, trophoblast invasion, and induction of tolerance towards the allogeneic embryo. The two immune cell subsets prevailing in decidua during pregnancy are uNK and monocytes/antigen-presenting cells with characteristic stage-dependent migration and distribution. Their recruitment into the decidua depends on the microenvironmental stimuli for chemoattraction. Characteristically, localized activation of inflammatory cytokines (TNF-*α*, IL-1, IL-6 [108], and IFN-*γ*) secreted by the recruited NK cells and Mo/Mph [109] occurs in the decidua around the time of embryo implantation. These cytokines influence the decidualization of DSCs and therefore determine the crosstalk between immune and nonimmune cells in the differentiating decidual milieu. Later on, the decidual niche is enriched in anti-inflammatory factors supporting maternal immune tolerance towards the embryo.

*(5) Immune Cell Migration into the Placenta*. Decidualization of the endometrial cells induces profound changes of their gene expression profiles and the secretion of bioactive mediators (hormones, transcription factors, cytokines, chemokines, selectins, integrins, tissue metalloproteinases, proteins, lipids, etc.) which are operational at every stage of placental remodelling [8, 9]. When stimulated by steroid hormones, the epithelial cells and DSCs produce chemokines (CCL2/MCP1, CXCL8/IL-8, CXCL10/IP-10, CX3CL1/fractalkine [110], CXCL10, and CXCL11 [111]) and cytokines (IL-6, IL-8, and IL-15) [32] and create an immune milieu in the decidua. Chemokines are chemoattractant cytokines that navigate the trafficking of leukocytes into organs in order to perform homeostatic immune surveillance or reach sites of inflammation [112]. Depending on the environmental (external/internal) stimuli, tissues encode combinations of chemokine patterns, which are specifically recognized by receptor expressing immune cells subsets [113, 114]. Cytokines and hormones, independently and in combination, can influence the expression of chemokines [110, 111, 115, 116], chemokine receptors [110], adhesion molecules, and their ligands [54] within different cells and tissues, such as leukocytes and the endothelium [114]. For example, LIF is reported to augment chemokine (MCP-1 and CXCL8) and cytokine (IL-6 and IL-15) expression in stromal cells [32], thus potentiating the effect of progesterone/progesterone and estradiol. Furthermore, TNF-*α* and IL-1*β* are shown to induce MCP-1 [116], whereas IL-1*β* stimulates expression of the chemokines MCP-1, CXCL8, CCL5/RANTES, CXCL2/MIP-2*α*, and CXCL3/MIP-2*β*, which are known to recruit monocytes/macrophages to tissues [115]. However, there might also be chemokines that are not regulated by hormones. An example is the chemokine CXCL12/SDF1, which is exclusively and highly expressed in decidual stromal cells but only scarcely expressed in epithelial cells. CXCL12 expression remains unchanged after progesterone treatment of stromal cells [110].

The chemokine repertoire of the decidua and placenta define the temporal recruitment of uNK cells [110] and monocytes [115] from the peripheral blood (Figure 3). The suggested mechanism implies that the progesterone and estradiol upregulate the chemokines CXCL10 and CXCL11 in the decidua [111], while progesterone is shown to upregulate their receptor CXCR3 on NK cells [110]. CXCL10 and CXCL11 along with CXCL12 and CX3CL1 create a specific environment in the decidua, which attracts CXCR3 and CXCR4 receptor expressing peripheral blood NK cells, to migrate into the decidua [110, 111]. Recruited locally, the peripheral blood NK cell subsets CD56^+^CD16^+^ and CD56^+^CD16^null^ are converted into decidual CD56^bright^CD16^null^ uNK by the tissue-specific microenvironment. The main role in this process is attributed to the decidual stromal cells, which contribute to the maintenance of uNK cells via TGF*β*1 signalling, which triggers their differentiation [117], and IL-15 signalling, which controls their proliferation [118]. Similarly to the factors mentioned before, the expression levels of TGF*β*1 and IL-15 in stromal cells are decidualization-dependent [32] and were found to progressively increase in decidual tissue sections from the secretory phase to the first trimester [118, 119]. Located in the decidua and differentiated accordingly, the uNK cells still express CXCR3 [111] and CXCR4 [120] chemokine receptors. They are chemoattracted to the vicinity of trophoblast cells that have been shown to spontaneously secrete CXCL12 [120] and CCL3/MIP-1*α* [121], but not CXCL11, CXCL10, and CXCL9 chemokines [120]. This profile suggests the utilization of CXCR4 [120] and CCR5 chemokine receptors by uNK cells for driving their migration towards trophoblasts invading the spiral arteries [120]. In this way, the uNK cells, which are enriched in the decidua close to the site of embryonic implantation, release factors and control the remodelling of spiral arteries [122] and trophoblast invasion [123–125].

Monocytes are another subset of immune cells that plays a role during pregnancy. They are sensitised to the combinatorial profile of chemokines expressed by DSCs that recruits them from the blood flow into the decidua. Thereafter, they interact with CXCL16 [126], MIP-1*α* [121], and chemokines released from trophoblasts and relocate to the proximity of foetal placental cells. Locally, monocytes are exposed to the placental environment (decidua and trophoblasts) and differentiate into antigen-presenting cells (APCs)—macrophages [127] or dendritic cells [128] with tolerogenic phenotype and function [129, 130]. APCs in the decidua demonstrate reduced allogeneic reactivity [128] and produce many immune factors, such as IL-10, IL-15 [127], and the chemokine MCP-1 [128], which play a role in immune suppression, differentiation, and recruitment of cell subsets. Altogether, the synchronized contact of immune, decidual, and trophoblast cells prompts their own temporal and cell-specific distribution, differentiation, and function, thus facilitating proper placental development and overall tissue homeostasis.

## 2. Immune Modulatory Properties of DSCs

The acceptance of a semiallogeneic embryo and the maintenance of immune tolerance during pregnancy alter leukocyte distribution and function in the maternal decidua. However, pregnancy hormones, the decidualization process, and the interactions of decidual and trophoblast cells with immune cells help to establish pregnancy-related immune homeostasis. This highlights the importance of intercellular communications in the implantation niche for the physiology of pregnancy in health and disease. DSCs might be the main operational immune modulators in placenta able to change lymphocyte function and suppress immune responses. Recent application of DSCs in clinical trials for treatment of steroid-resistant graft-versus-host disease shows the strong immune suppressive ability of DSCs [131].

### 2.1. DSCs and NK Cells

NK cells belong to the innate immunity and divide into noncytotoxic NK cells (CD56^bright^CD16^low/neg^KIR^neg^) and cytotoxic NK cells (CD56^dim^CD16^+^) [132]. The cytotoxic NK cells lyse the virus-infected and tumour cells by secreting Granzyme A and Perforin. NK cells circulate in peripheral blood and through the secondary lymphoid organs but are also residing in tissues and organs such as the skin, lung, liver, and uterus [132, 133]. In the uterus NK (uNK) cells are the predominant immune population and represent approximately 70% of all leukocytes [134]. uNK cells are noncytotoxic [135] and have nonimmune functions during the first trimester of pregnancy, including tissue remodelling, neoangiogenesis, and control of trophoblast invasion [125]. Two mechanisms of uNK cell enrichment have been suggested [136, 137]. As previously described, NK cells migrate from the peripheral circulation to the decidua where they undergo a modification of their function. In addition, NK cells might appear as a result of differentiation of tissue resident haematopoietic CD34^+^ precursor cells [137]. The presence of haematopoietic precursor cells (CD45^+^CD34^+^) that give rise to uNK cells was first described by the group of Vacca [137]. These precursor cells are found to express membrane receptors specific for NK cell mitogen IL-15-CD122 and mIL-15 as well as NK specific transcription factors E4PB4 and ID2. Therefore, CD34^+^ cells in decidua are considered to be NK lineage restricted. Their commitment is demonstrated by* in vitro* experiments in which stimulation of CD34^+^ cells with a combination of stem cell factor, FMS-like tyrosine kinase ligands, IL-7, IL-15, and IL-21, leads to their differentiation into mature NK cells [137]. Moreover, coculturing with DSCs causes CD34^+^ cells to differentiate into mature NK cells that secrete IL-8 [137]. It has been shown that DSCs can suppress the proliferation of IL-15 activated NK cells [138] and can inhibit their Granzyme A and Perforin synthesis. As a result, the NK cell cytotoxicity has been impaired and decreased lysis of erythroleukemia (К562) and melanoma (FO1) target cells has been observed [138]. This inhibition on NK cell proliferation and cytotoxicity is mediated by the immune suppressive factors PGE2 and indoleamine 2,3-dioxygenase (IDO), which are secreted by DSCs. However, a bidirectional interaction between DSCs and NK cells is necessary for the induction of tissue-specific cell functions. For example, Croxatto et al. point out that interferon *γ* (IFN*γ*) produced by NK cells is necessary for the activation of* ido* transcription in DSCs [138]. Altogether, the summarized literature suggests that the cells from the decidual niche create specific microenvironment that modulates cell functions.

### 2.2. DSCs and Antigen-Presenting Cell Populations

Various APCs are observed in the human decidua during pregnancy. The majority of these are macrophages (Mph), while the number of mature dendritic cells (DCs) is much smaller [139]. Besides APCs with a classical profile, there are also cells simultaneously expressing markers characteristic of Mph and DCs [140]. This DC/Mph phenotype suggests either that these cells are in intermediate differentiation stages or that they constitute a separate APC subpopulation. In the decidua, these DC/Mph phenotype cells are found in the vicinity of the spiral arteries and are often clustered with uNK cells [140]. In contrast, the mature DCs are located close to lymphatic vessels and in contact with T lymphocytes. Although decidual APCs are in contact with different immune cells, their function is not completely elucidated. It has been hypothesised that they do not act as classical APCs to activate allospecific effector cells. Still, the function of DCs, and in particular whether they act as immune activators or suppressors, is unclear.* In vitro* experiments demonstrate that DC/Mph phenotype cells isolated from the decidua activate allogeneic lymphocytes to a twofold lesser extent in comparison to mature DCs [139].

The state of maturity of DCs is a major control mechanism regulating the antigen-specific immune responses. Croxatto et al. showed that DSCs modulate the DC maturation process [138]. In the presence of DSCs, the differentiation of blood monocytes to immature DCs (CD14^+^CD1a^+^) via IL-4 and GM-CSF cytokines is inhibited by 50–96%. Although phenotyped as fully differentiated, the rest of the cells remain weak activators or, probably, inhibitors of allogeneic T lymphocytes proliferation [138]. In addition, DSCs inhibit the differentiation of immature DCs into mature DCs via the secretion of MIC-1 (Macrophage Inhibitory Cytokine-1) by DSCs. Furthermore, DSCs block the expression of CD25, CD83, and CD86 receptors, which could explain the observed weaker activation potential of DCs [141]. Altogether, these observations demonstrate that DSCs support decidua-specific microenvironment and modify the functions of infiltrating APCs.

## 3. DSC Involvement in Reproductive Pathological Conditions

**Figure 4:**
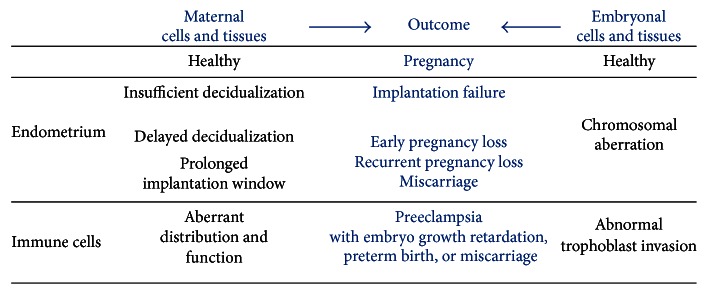
Pregnancy complications due to maternal and/or embryonic tissue functional aberrancy.

The implantation of the blastocyst is the critical event in human pregnancy and is strongly dependent on the physiological state of the blastocyst as well as the uterine lining [142, 143]. Evolution has endowed the endometrium with the ability to evaluate embryo quality [144]. Either the implantation of a low-quality embryo does not occur or the embryo is rejected soon after implantation [95]. Apart from this natural protective mechanism, the pathology-related implantation failure occurs also due to an inappropriately differentiated endometrium (Figure 4). Therefore, any disturbance in the decidualization programme and/or maternal-foetal crosstalk could cause pregnancy complications such as implantation failure, pregnancy loss, or development of a chromosomally abnormal embryo. The adequacy of endometrial functionality for pregnancy progression can be estimated by the fraction of abnormal embryos from miscarried pregnancies. While women with sporadic spontaneous abortions miscarry chromosomally normal embryos in 40–50% of the cases [95], this percentage is almost twice higher among women with recurrent pregnancy loss [145]. The higher rate of normal embryos loss, together with the lower rate of live births in consequent pregnancies in the patients with recurrent pregnancy loss, strongly suggests that endometrial dysfunction is the more probable cause for abortion in these cohorts [145].

In this sense, the precise coordination and synchronization of the factors related to decidualization are critical for the success of pregnancy. The change in expression and/or function of one particular factor might affect the whole network of factors, abrogate tissue homeostasis, and cause reproductive disorders. Indeed, studies of the endometrium of women with reproductive disorders have demonstrated dysregulation of gene [146, 147] and protein [148, 149] expression profiles. However, the number of the dysregulated genes varies among different studies depending on the applied statistical methods [146], on the investigated phase of the menstrual cycle [150], and the type of reproductive disorder [146, 147]. Moreover, for a given pathology, no clear relationship between the gene and protein expression profiles has been observed [148]. This suggests that the effect of the pathology occurs at the level of posttranscriptional modifications. This is consistent with investigations of miRNA expression, which reveal changed profiles in some pathological conditions [151, 152].

Investigations of whole endometrial biopsies also demonstrate dysregulation in gene and protein expression but do not reveal what are the affected cell subtypes. The analyses of signal transduction pathway components have demonstrated a relationship between the reproductive disorders and the impairment of progesterone signalling in the endometrium [148, 150, 153], which implies stromal cell dysfunctions. In addition, the involvement of other signalling pathways tightly connected to decidualization has also been suggested to contribute to pathological conditions. These pathway components include Dickkopf homolog1, FOXO1A, and TGF*β*2 and the receptors for PGE2, IL-1, LIF, and gp130 [150, 153]. Indeed, a growing number of reports attribute DSC dysfunction to a variety of reproductive diseases and pregnancy complications. Notably, decidual stromal cells isolated from patients with reproductive disorders demonstrate an aberrant* in vitro* decidualization as evaluated by lower PRL and/or IGFBP-1 expression levels in comparison to healthy controls. Impaired differentiation of stromal cells has been observed in pathologies with different aetiology, namely, endometriosis (EOS) [154, 155], recurrent pregnancy loss (RPL) [156], and antiphospholipid syndrome (APLS). EOS is defined as an oestrogen-dependent, progesterone-resistant gynaecological condition, manifested by endometrial lesions developing in the peritoneal cavity [157]. RPL is characterized by three or more consecutive abortions [158], while APLS is marked by the levels of circulating antiphospholipid autoantibodies and symptoms of vascular thrombosis and thrombocytopenia. However, despite these differences between the pathologies, all of them are accompanied by high infertility rate and reproductive complications, possibly predefined by the described impairment in the decidualization of DSCs. The case of APLS strongly supports this hypothesis. It has been accepted that the thrombotic events in the maternal as well as foetal-maternal circulation are the main cause for pregnancy complications in APLS. But an impairment of decidualization, as well as vasculature development in the implantation niche, has also been suspected [159]. The assumption is confirmed by the observation that the antiphospholipid autoantibody 2-glycoprotein-I decreases the expression of PRL and IGFBP-1 by ESC isolated from women with APLS in response to* in vitro* decidualization [71]. This finding is confirmed* in vivo* as well by the observation that the levels of PRL and IGFBP-1 transcripts in endometrial biopsies of RPL patients with an accompanying APLS are decreased and correlated to an increased rate of miscarriages in the group compared to the cohort of RPL without APLS [159]. The altered timing and amplitude of the decidualization response developed by the impaired stromal cells function or pathology-related environmental factors, such as circulating autoantibodies, reveal their correlation to the clinical manifestation of the investigated diseases. For example, in EOS, the level of estradiol, as well as enzymes involved in its synthesis, is found to be increased in ESCs [155, 160]. The ability of stromal cells to autonomously synthesize oestrogen [161], in addition to their progesterone resistance [162, 163], impairs decidualization. While in the endometriotic patients the decidualization process is incomplete, in RPL patients the endometrial transformation is significantly delayed [156, 164]. The molecular basis of decidual dysfunction in RPL lies in the impaired dynamics and expression of proinflammatory and implantation factor, such as IL-33 [164] and prokineticin-1 [156]. IL-33 regulates the decidual receptivity factors during the implantation window, while prokineticin-1 is involved in angiogenesis and control of decidualization and implantation processes. Both factors show delayed expression in* in vitro* decidualized DSCs; hence the resulting* in vivo* outcome is a prolonged implantation window, increased decidual receptivity, and impaired selective functions of decidua in RPL patients.

In addition to the dysregulated factors mentioned above, a number of mitogens and chemoattractants for immune cells are also affected. For example, DSCs isolated from women with polycystic ovary syndrome have increased expression of IL-6 and IL-8 in response to decidualization in comparison to DSCs from healthy individuals. Conditioned medium from these cells correspondingly increases the chemoattraction of CD14^+^ monocytes and CD4^+^T cells [165]. Conversely, IL-11 production is lower in ESCs from infertile compared to fertile women when their ESCs are subjected to* in vitro* decidualization [166]. This suggests that the deficiency of this cytokine in the case of infertility might be a sign of improper or abrogated decidual differentiation.

In reproductive disorders, it has been observed that chemoattracting molecules are overexpressed in DSCs. These elevated levels are often induced and promoted by the immune cells that are recruited in the decidua [167]. Thus the feedback of interactions between factors released by the decidual and immune cells is impaired, which disrupts the temporal and spatial distribution of immune cells and pregnancy homeostasis.

The significance of the cytokines, chemokines, and their receptors expressed at the foetal-maternal interface for the success of pregnancy is confirmed by studies of endometrial biopsies from patients with reproductive disorders. The results reveal a reduction in LIF, its receptor LIFR, and the signal transducing chain gp130 in patients with unexplained infertility [168], endometriosis [169–171], and recurrent implantation failure [172]. By contrast, in preeclampsia, LIF is increased [173]. However, signalling downstream of LIF is abrogated, because the corresponding transcription factor STAT3 and its phosphorylated, active form are reduced [174, 175]. Collectively, loss of LIF signalling has been established in patients with variable pathologies, such as preeclampsia, unexplained infertility, endometriosis, and recurrent implantation failure, indicating its importance for the maintenance of reproductive functions.

Other pregnancy factors, related to leukocyte homing to the foetal-maternal interface, are also dysregulated in patients with different reproductive pathologies. Briefly, impaired expression of CXCR4 [146] and enhancement of IL-15 in women with implantation failure [172] and augmented expression of CXCL13, IL-8, MCP-1, and *α*_v_ *β*_3_ in endometriosis [176–179] as well as increased expression of the intercellular adhesion molecule-1 (ICAM-1) in the placenta of foetal growth restricted pregnancies [180] have been observed. These data suggest that the foetal-maternal interface in complicated pregnancies is enriched in chemoattracting molecules and their corresponding receptors, which is likely to be accompanied by an abnormal influx of immune cells into the decidua.

The relationship between the impairment of molecules regulating immune cell migration and the presence of immune cells in the endometrium has been shown in women with recurrent implantation failure. A significantly higher number of CD56^+^ uNK cells in the midsecretory endometrium are observed in the diseased group [181], which correlates with a significantly higher IL-15 expression in the endometrial stroma [172] in comparison to healthy controls. An enrichment of uNK cells in the decidua is also observed in other reproductive disorders [182, 183]. Interestingly, Kuroda et al. [183] found a reverse dependency between the number of CD56^+^ cells accumulating in the subluminal decidua and the ability of primary ESC cultures to decidualize* in vitro*. The results show that the more the NK cells are present in the tissue, the weaker the decidualization evaluated by the expression of PRL and IGFBP1 in stromal cells is. The authors observed decreased mRNA levels of the enzyme 11*β*HSD1 in ESC decidualized* in vitro*, which is consistent with the increased presence of CD56^+^ cells in the corresponding tissue. This enzyme is involved in the synthesis of cortisol, a potent inhibitor of uNK cell cytotoxicity [184]. Hence, it is possible that cortisol deprivation caused by 11*β*HSD1 insufficiency in the stromal cells leads to the enrichment of activated NK cells, linked to low fertility status of the patients. Support for this hypothesis is given by the group of Giuliani et al. [182]. The authors investigated the distribution of the CD56 expressing uNK subpopulations and their activation status by the evaluation of the expression of CD16 and the activation marker NKp46. Contrary to the previous report, in this study a significant difference of the number of CD56^+^ cells was not found. However, the authors [182] found a correlation between the increased number of activated uNK and the investigated pathologies (unexplained infertility, unexplained RPL, and endometriosis) [182]. Altogether, further investigations will be needed to confirm a connection between the number/activation of uNK cells and endometrial dysfunctions.

The APCs are the second most prevalent immune cell population in the uterine lining, involved in embryo recognition and tolerance. Tolerogenic cells (DC-SIGN^+^DC and CD163^+^Mph), mature CD83^+^DC, and APCs with a DC/Mph phenotype have been described [134, 139, 140, 185]. The maintenance of a specific phenotype, distribution, and functional status among the APC populations is essentially dependent on the microenvironment. In many pregnancy complications, abnormal APC activation and distribution are suggested. The Mph, defined as CD14^+^ cells, in third-trimester decidua of women with preeclampsia were found to be decreased using immunohistochemical analysis [186], although this observation was not confirmed in another study where a bigger cohort was analyzed by FACS analysis [187]. In addition, no difference in CD68^+^CD14^−^ and CD68^+^CD14^+^ Mph density and distribution was observed in the decidua of patients with preterm birth with or without preeclampsia [188]. Furthermore, another study elucidates a general change of CD14^+^/CD163^+^ or DC-SING/CD163^+^ (CD14^+^ and CD163^+^) Mph in the decidua basalis of women with preterm birth and preeclampsia in comparison with patients without preeclampsia [185]. In conclusion, more investigations will be required to reach a consensus on the role of APCs in directing the immune response to tolerance or activation in pregnancy complications.

## 4. Conclusion

Hormonally controlled differentiation of ESCs into DSCs is decisive for transforming the uterine mucosa/decidua into a receptive implantation site, for performing selection, and for achieving immune acceptance of the allogeneic foetus. The summarized literature emphasizes the important contribution of decidual stromal cells to the microenvironment and their direct or indirect influence on immune cell recruitment, distribution, and function, on tissue remodelling, and on placenta formation. This clarifies the association between impairment in the timing and the state of differentiation of DSCs with the occurrence of severe pathological conditions, such as endometriosis, RPL, and APLS, all of which are with poor prognosis for human reproduction.
